# Safetxt: a pilot randomised controlled trial of an intervention delivered by mobile phone to increase safer sex behaviours in young people

**DOI:** 10.1136/bmjopen-2016-013045

**Published:** 2016-12-23

**Authors:** Ona L McCarthy, Rebecca S French, Paula Baraitser, Ian Roberts, Sujit D Rathod, Karen Devries, Julia V Bailey, Phil Edwards, Kaye Wellings, Susan Michie, Caroline Free

**Affiliations:** 1 The London School of Hygiene and Tropical Medicine, London, UK; 2 Kings College London, London, UK; 3 University College London, London, UK

**Keywords:** SEXUAL MEDICINE, INFECTIOUS DISEASES

## Abstract

**Objective:**

To test the procedures proposed for a main trial of a safer sex intervention for young people delivered by mobile phone text message (‘safetxt’).

**Design and setting:**

Pilot randomised controlled trial. Participants were recruited through sexual health services in the UK. An independent online randomisation system allocated participants to receive the safetxt intervention or to receive the control text messages (monthly messages about participation in the study). Texting software delivered the messages in accordance with a predetermined schedule.

**Participants:**

Residents of England aged 16–24 who had received either a positive chlamydia test result or reported unsafe sex in the last year (defined as more than 1 partner and at least 1 occasion of sex without a condom).

**Intervention:**

The safetxt intervention is designed to reduce sexually transmitted infection in young people by supporting them in using condoms, telling a partner about an infection and testing before unprotected sex with a new partner. Safetxt was developed drawing on: behavioural science; face-to-face interventions; the factors known to influence safer sex behaviours and the views of young people.

**Outcomes:**

The coprimary outcomes of the pilot trial were the recruitment rate and completeness of follow-up.

**Results:**

We recruited 200 participants within our target of 3 months and we achieved 81% (162/200) follow-up response for the proposed primary outcome of the main trial, cumulative incidence of chlamydia at 12 months.

**Conclusions:**

Recruitment, randomisation, intervention delivery and follow-up were successful and a randomised controlled trial of the safetxt intervention is feasible.

**Trial registration number:**

ISRCTN02304709; Results.

**Strengths and limitations of this study**

- This pilot trial demonstrated that the proposed procedures for a main trial of the safetxt intervention are feasible.
- Follow-up was high compared with a similar trial collecting sexually transmitted infection outcomes. Additional strategies to increase follow-up are needed.
- The safetxt intervention was acceptable to recipients.

## Introduction

### Background of the problem

The prevalence of genital chlamydia infection (hereafter referred to as ‘chlamydia’) in the UK is highest among people aged 16–24, estimated to be 3.1% for women and 2.3% for men.^1^ Long-term adverse health effects of chlamydia infection may include ectopic pregnancy and subfertility, especially in those with repeated infections.^1^ ^2^ Reinfection among young women treated for chlamydia is particularly high.^3^ Skills such as negotiating condom use, notifying a partner about an infection and testing can reduce the risk of infection. However, young people can lack the confidence and skills needed to adopt safer sex behaviours.^4^

### Mobile phones for intervention delivery

Phone ownership in the UK is high, with an estimated 93% of adults owning a personal phone and 90% of people aged 16–24 owning a smart phone.^5^ Among young people in the UK, mobile phones have become constant companions; 59% say that their mobile phones would be the device that they would miss the most if taken away and almost half (48%) report checking their phones within 5 min of waking.^5^ Mobile phones therefore have the potential to deliver widely accessible and inexpensive health behaviour support. With sensitive health topics such as sexual health, mobile phones can offer convenience and privacy.

### Existing research

Interventions delivered by mobile phone can be effective in improving a range of behaviours such as smoking cessation, adherence to medication and contraceptive uptake.^6–14^ There is some evidence that mobile phone support can increase safer sex behaviours; however, high-quality trials are needed to reliably establish effects.^15^ A trial evaluating an intervention consisting of eight safer sex text messages, aimed at increasing knowledge and perceived behavioural control, reinforcing safer sex behaviours and changing attitudes, found that intervention participants were more likely to report using condoms with new partners in the previous 3 months compared with controls who received text messages not about safer sex (OR 2.2, 95% CI 1.1 to 4.2). However, this trial found little evidence for an intervention effect on always using condoms in the previous 6 months (OR 0.8, 95% CI 0.5 to 1.4).^16^ An intervention consisting of 14 ‘short and catchy’ text messages providing advice and information about sexually transmitted infection (STI), increased STI testing in women compared with control participants who did not receive the messages (OR 2.51, 95% CI 1.11 to 5.69).^17^ A trial of an intervention aimed at retesting following a positive chlamydia test result found a fourfold increase in testing in participants that received text message reminders versus controls that received the standard advice from a clinician (RR 4.5, 95% CI 1.05 to 19.22).^18^ None of these trials had a low risk of bias or targeted partner notification. The interventions evaluated in these trials included up to three behaviour change techniques (intervention components aimed at influencing behaviour).^19^

### Intervention development

We developed an intervention delivered by text message to target condom use, partner notification and STI testing. We developed the intervention based on: behavioural science;^19^ ^20^ the content of effective face-to-face safer sex interventions;^21^ the factors known to influence safer sex behaviours;^4^ ^22^ the views of 82 young people collected in focus group discussions^23^ ^24^ and a questionnaire completed by 100 people aged 16–24.^23^ ^24^ In interviews with 16 participants, young people reported that the intervention tone, frequency and content were acceptable.^25^ Our pilot trial builds on this successful intervention development work.^23^

## Methods

The objective of this pilot trial was to test the procedures proposed for a main trial of the safetxt intervention. We aimed to recruit and randomise 200 participants within 3 months and achieve 80% response at each follow-up point. There were no changes to the methods after the trial started.

### Eligibility criteria

Residents of England aged 16–24 who had received either a positive chlamydia test result or reported unsafe sex in the last year (defined as more than one partner and at least one occasion of sex without a condom), were literate in English and who owned a personal mobile phone were eligible.

### Recruitment

We recruited participants from seven sexual health services located in inner city Manchester, South London, Cambridgeshire, Norfolk, Maidstone and Hull. Recruitment staff enrolled participants at the service or, with their permission, gave the telephone numbers of eligible people to the trial manager at LSHTM who contacted them by phone for recruitment. We gave all potential participants detailed verbal and written information, the opportunity to ask questions and time to consider their participation. For participants recruited by telephone, the trial manager emailed or texted the link to the web-based information sheet and participants provided informed consent by completing the form on the secure study website. We recorded the number of participants that were assessed, were eligible and declined.

### Treatment groups

Our texting software automatically delivered the intervention or control messages to the mobile phone number that participants provided at enrolment. The software delivered the messages in accordance with a predetermined message schedule. All participants had the option of choosing embargoed times when they would not receive messages.

#### The safetxt intervention

The safetxt intervention includes 12 behaviour change techniques and involves the functions education, enablement and incentivisation.^19^ ^20^ ^23^ It consists of short, non-judgmental text messages designed to reduce STI in young people by supporting them in using condoms, telling a partner about an infection and testing before unprotected sex with a new partner. There are four intervention message sets, tailored to gender and infection status at enrolment sent over 12 months: women-positive (63 messages), men-positive (61 messages), women-negative (51 messages) and men-negative (49 messages). Around half of the messages in each set are delivered in the first month: 59% (37/63) of the women-positive, 59% (36/61) of the men-positive, 49% (25/51) of the women-negative and 49% (24/49) of the men-negative messages (see online supplementary file 1). The messages are delivered to each participant according to the set schedule, that is, each participant starts at message #1 and receives the message set consecutively. Participants who had a positive STI test result at the time of enrolment received additional messages during the first week after randomisation on obtaining and taking treatment and notifying partners about their infection. See Free 2016 for a full details of the development of safetxt.^23^

10.1136/bmjopen-2016-013045.supp1 supplementary file

#### Control

Participants allocated to the control group received 13 messages, starting at the time of randomisation, spaced 30 days apart. The control messages reminded them of their participation with the aim of keeping them engaged in the trial. The control messages contained no behaviour change techniques.

See online supplementary file 1 for the intervention and control message frequency and online supplementary file 2 for example intervention and control messages.

10.1136/bmjopen-2016-013045.supp2 supplementary file

### Outcomes

#### Primary outcomes

The coprimary outcomes were the recruitment rate and the completeness of follow-up for assessment of the proposed primary outcome for the main trial, cumulative incidence of chlamydia at 12 months.

#### Process outcome data

We collected process outcome data at 1 and 12 months postrandomisation.

#### Acceptability of the intervention

The 1-month questionnaire also collected data on participants' views of the messages. We gathered participant views at 1 month because around half of the messages were delivered within this period and we wanted to maximise recall. An additional measure of acceptability is the number of participants allocated to the intervention that requested for the messages to stop.

### Sample size

The pilot trial was designed to estimate the likely follow-up rate at 12 months. If loss to follow-up in the main trial was 20%, a pilot trial of 200 participants would estimate the loss to follow-up with a precision of 6% (ie, a 95% CI of 14% to 26%).

### Protection against bias

#### Randomisation

An independent online randomisation system (sealed envelope) generated the 1:1 allocation sequence, assuring allocation concealment. The sequence was stratified by site using random permuted block sizes of 2, 4 and 6. No one involved in the research was aware of the block sizes. The system randomised participants immediately after the baseline data were entered.

#### Blinding

As this is a behavioural intervention, participants were aware of their treatment allocation and therefore were non-blinded. The trial manager required access to treatment allocation in order to identify intervention participants for qualitative interviews.^25^ Laboratory staff assessing chlamydia infection and researchers assessing the outcomes were blinded to treatment allocation. Research staff performing the statistical analysis were blinded to treatment allocation. Data were double entered, with one researcher blinded to allocation. The treatment allocation variable in the data set was coded 1 or 2 and this was kept undisclosed to two of the three research staff performing the analysis (one was the trial manager) until the full analysis was complete.

### Data collection and follow-up procedures

We assessed numbers recruited by the number randomised during the 3-month time-period. We assessed follow-up response by numbers completing the questionnaire at 1 and 12 months and numbers returning a chlamydia test sample at 3 and 12 months. We also collected clinic data for tests within the 12-month follow-up period.

We recruited participants face-to-face in clinics and by telephone, depending on the service. We collected baseline data by paper questionnaire in face-to-face recruitment. At the Cambridge service, staff assessed eligibility when providing positive chlamydia test results over the phone. With the potential participant's agreement, staff at the service provided their telephone number to the Project coordinator, who then contacted the participant to enrol them and collect baseline data over the phone. Recruitment staff who obtained informed consent and administered the baseline questionnaire entered the baseline data onto a secure online trial database system. If the test result was pending when the participant was enrolled, recruitment staff entered the baseline data onto the system within 24 hours of the test result. All participants enrolled by telephone referral had received a positive chlamydia test result and the trial manager entered their data on the day that they were recruited.

We requested all outcome data by post in the first instance and followed up non-responders by email and phone. At 1 month, we sent an unconditional £5 incentive with the questionnaire. At 3 months, we sent £5 unconditional incentive and a postal chlamydia test kit. Men were sent a urine test (sensitivity 98.1%, specificity 99.5%) and women were sent a vulvo-vaginal swab (sensitivity 94.1%, specificity 99.7%). At 12 months, we sent an unconditional £10 incentive with the 12-month questionnaire and test kit. Participants were given the option of returning their questionnaire by post or completing the questionnaire on the online database system. Participants were told that they would receive £20 for returning the test sample when they received the test kits. Participants sent the test kit in a prepaid envelope directly to the laboratory. Recruitment sites provided clinic data for positive tests during participants' involvement in the trial (participants consented to this at enrolment). Our approach to developing the follow-up procedures involved identifying the effective methods to increase follow-up from systematic reviews, testing prototype procedures and consulting with the target group regarding the acceptability of the procedures.^26^ We assessed the proportion of messages successfully delivered using our trial database metrics.

### Analysis

We conducted all analyses in Stata V.14. We conducted all analyses once, at the end of the trial. We calculated the follow-up response as a proportion and report the 95% CI.

See online supplementary file 3 for the Pilot trial protocol.

10.1136/bmjopen-2016-013045.supp3 supplementary file

## Results

**Figure 1:**
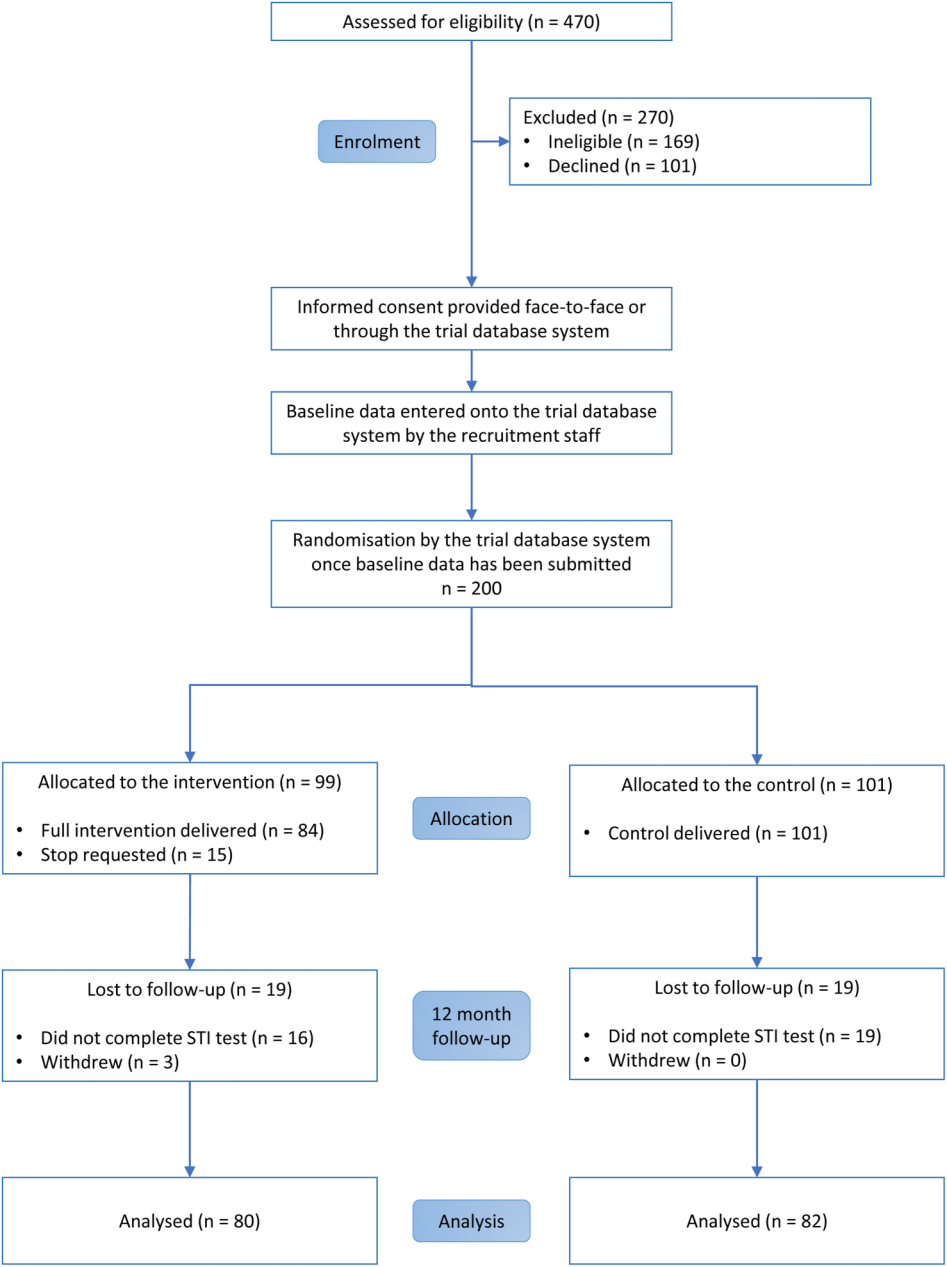
CONSORT diagram.

We assessed 470 people for eligibility, of which 169 were ineligible and 101 declined. Sixty-six per cent of eligible participants joined the trial (200/301). We randomised 200 participants between 9 September 2013 and 26 November 2013 (figure 1).^23^ Ninety-nine participants were allocated to the intervention and 101 were allocated to the control group. We conducted follow-up between October 2013 and February 2015. There were some differences between groups in ethnicity and baseline infection (table 1). Ninety-two per cent of participants provided 1-month questionnaire data (183/200), 86% provided a chlamydia test sample at 3 months (171/200) and 82% provided 12-month questionnaire data (163/200).^26^ Fifteen participants requested the intervention messages to stop (15%, 15/99).

**Table 1:** Baseline demographic and sexual behaviour characteristics

Percentages are of group total unless specified	Control group (%)	Intervention group (%)
Gender		
Male	31/101 (30.69)	29/99 (29.29)
Female	70/101 (69.31)	70/99 (70.71)
Age		
Mean (SD)	20.60 (2.39)	20.39 (2.42)
16–19 years	33/101 (32.67)	36/99 (36.36)
20–24 years	68/101 (67.33)	63/99 (63.64)
Ethnicity		
White	55/101 (54.46)	59/99 (59.60)
Black	32/101 (31.68)	21/99 (21.21)
Asian	0/101 (0.0)	2/99 (2.0)
Chinese	0/101 (0.0)	0/99 (0.0)
Other	14/101 (13.86)	17/99 (17.17)
Refused/missing	0/101 (0.0)	0/99 (0.0)
Sexual orientation		
Heterosexual	83/101 (82.18)	88/99 (88.89)
Gay or lesbian	5/101 (4.95)	3/99 (3.03)
Bisexual	10/101 (9.90)	5/99 (5.05)
Refused/missing	3/101 (2.97)	3/99 (3.03)
STI infection at baseline		
No infection	53/101 (52.48)	58/99 (58.59)
Chlamydia-positive	42/101 (41.58)	35/99 (35.35)
Gonorrhoea/NSU	5/101 (4.95)	5/99 (5.05)
Chlamydia/gonorrhoea/ NSU diagnosis	1/101 (0.99)	1/99 (1.01)
Sexual behaviour		
Condom use at last sex	35/101 (34.65)	32/99 (32.32)
Condom use at last sex with someone new	52/101 (51.49)	48/99 (48.48)
Testing prior to last sex with someone new	37/101 (36.63)	32/99 (32.32)
Partner testing at last sex with someone new*	12/101 (11.88)	11/99 (11.11)
Number of sexual partners in last 12 months		
0	0/101 (0.0)	0/99 (0.0)
1	9/101 (8.91)	6/99 (6.06)
2+	92/101 (91.09)	93/99 (93.94)

*Participants were asked “The last time you had sex with someone new, did they get tested for sexually transmitted infections before you had sex?”.

### Primary outcomes

We recruited 200 participants within 3 months and we achieved 81% follow-up completion (162/200, 95% CI 74.86 to 86.19) for cumulative incidence of chlamydia at 12 months^23^ ^26^ (table 2).

**Table 2:** Primary outcome data

Follow-up	Control group n/N (%)	Intervention group n/N (%)	Follow-up n/N (%, 95% CI)
12-month follow-up for cumulative incidence of chlamydia (trial test kits+clinic data)	82/101 (81.19)	80/99 (80.81)	162/200 (81.0, 74.86 to 86.19)
12-month follow-up for trial test kits	80/101 (79.21)	80/99 (80.81)	160/200 (80.0, 73.78 to 85.31)

### Process outcome data

Process outcome data for intervention recipients are reported in table 3. All intervention messages were sent successfully from our system. Over 97% of the intervention messages were successfully delivered to participants' phones, as confirmed by the gateway. At 1 month, 82% (73/89) of participants in the intervention group reported reading all messages and at 12 months, 74% (57/77) reported reading all messages. At 12 months, two participants in the control group reported reading messages sent to other trial participants.

**Table 3:** Process outcome data for intervention recipients

	Month 1 n/N (%)	Month 12 n/N (%)
Number of text messages read		
All	73/89 (82.02)	57/77 (74.03)
Some	10/89 (11.24)	18/77 (23.38)
None	6/89 (6.74)	2/77 (2.60)
If anyone read messages sent to the participant	24/92 (26.09)	19/80 (23.75)
If yes, how the participant felt about this		
Happy	7/24 (29.17)	8/19 (42.11)
Unhappy	2/24 (8.33)	2/19 (10.53)
OK	15/24 (62.50)	9/19 (47.37)

### Acceptability of the intervention

Participants' views about the acceptability of the intervention at 1 month are reported in table 4. Over 80% of intervention recipients reported that the text messages ‘made me think’ (83%, 71/86), were ‘respectful’ (88%, 76/86) and ‘easy to understand’ (95%, 81/85). Thirty-eight per cent (32/85) of intervention recipients reported that the messages ‘made me take action’. About two-thirds of intervention recipients reported that the messages ‘were from someone they could trust’ (66%, 57/86) and ‘came at the right time of day’ (65%, 56/86). Thirteen per cent (11/85) thought that the messages talked down to them. Twenty-four per cent (20/85) thought that there were too many messages and about 20% (17/85) thought that there were too few.

**Table 4:** Intervention group participant views regarding the messages at month 1

	Agree n/N (%)	Unsure n/N (%)	Disagree n/N (%)
The text messages made me take action	32/85 (37.65)	32/85 (37.65)	21/85 (24.71)
The text messages made me think	71/86 (82.56)	7/86 (8.14)	8/86 (9.30)
The text messages were from someone I could trust	57/86 (66.28)	21/86 (24.42)	8/86 (9.30)
The text messages were respectful	76/86 (88.37)	8/86 (9.30)	2/86 (2.33)
The text messages talked down to me	11/85 (12.94)	15/85 (17.65)	59/85 (69.41)
The text messages were easy to understand	81/85 (95.29)	3/85 (3.53)	1/85 (1.18)
There were too few text messages each day	17/85 (20.0)	18/85 (21.18)	50/85 (58.82)
There were too many text messages each day	20/85 (23.53)	17/85 (20.0)	48/85 (56.47)
I would have liked the text messages to stop sooner	12/86 (13.95)	25/86 (29.07)	49/86 (56.98)
The text messages came at the right time of day	56/86 (65.12)	21/86 (24.42)	9/86 (10.47)

## Discussion

### Principal results

The pilot trial demonstrated that the proposed procedures for a fully powered trial were successful. We fully recruited to target and achieved 81% follow-up for our proposed primary outcome for the main trial, cumulative incidence of chlamydia at 12 months. Participants' views suggested that the intervention is acceptable. Ninety-seven per cent of messages sent were successfully delivered to participants' phones.

### Strengths and limitations

In the pilot trial, we achieved acceptable follow-up, allocation was concealed and laboratory staff and those analysing data were blinded to allocation. Only one researcher who double entered the data was blinded to allocation. While the trial manager was non-blinded, the risk of bias associated with this was low as the intervention was delivered by the automated texting software, not by the trial manager. We present a complete case analysis of the process outcomes and participants' views about the acceptability of the intervention. The main trial will include sensitivity analyses in relation to missing data, which could reveal that participants lost to follow-up were less likely to read the messages and less likely to find them acceptable. Given the small sample and the large number of variables assessed, baseline characteristics were reasonably well balanced. A larger sample size in a main trial would allow for better balance between the arms. The pilot trial was not powered for behavioural or STI outcomes.

### Comparisons with other studies

The pilot trial achieved a higher follow-up response than a pilot trial of a sexual health website intervention for young people (‘Sexunzipped’). This trial achieved 45% follow-up using for chlamydia postal test kits and 72% for self-reported data at 3 months.^27^ Our approach to developing the follow-up procedures^26^ was similar to the approach used in the smoking cessation txt2stop pilot and main trial, which achieved high follow-up response.^6^ ^28^ Additional strategies to increase follow-up will be developed for the main trial, such as providing incentives to recruitment sites that have the highest proportion of participants completing follow-up.

Effective behavioural support interventions delivered by mobile phone messaging are inexpensive and can be delivered with high fidelity at scale.^6^ ^29^ ^30^ An effective mobile phone messaging intervention shown to decrease STI would be inexpensive to deliver at scale with high fidelity. In contrast, face-to-face STI interventions often consist of multiple sessions,^31–34^ which require ongoing staff training to ensure fidelity in delivery and much greater resources than safetxt.

### Implications for the main trial

We are currently conducting a main trial of the safetxt intervention, informed by a number of findings from the pilot trial to evaluate the effects of the intervention. The main trial sample size is powered to produce a precise estimate for the effect of the intervention on cumulative incidence of STI at 12 months. We will use the follow-up procedures used in the pilot trial and we will have enough staff for all those entering data to be blinded to allocation. In the pilot trial, 75% of participants that tested positive during the study also tested positive at enrolment (18/24). In the main trial, only people that test positive at the time of enrolment are eligible, enabling the trial to achieve the estimated event rate with a smaller sample size. As the target group in the main trial differs from the pilot trial, we will monitor recruitment to enable us to develop strategies to recruit any groups that are under-represented. In order to achieve a more demographically diverse sample, we are also recruiting from clinics with greater numbers of male clients that report having sex with men. The main trial will include a mediation analysis of the hypothesised mechanisms of action of the intervention, for example, we will investigate if the intervention increases condom use self-efficacy and self-efficacy in telling a partner about an infection.

The postal test kits and monetary incentives for returning them were not part of usual care during the pilot trial, but part of the pilot trial procedures and delivered to participants in the intervention and the control group. In the main trial, we are sending postal test kits at 12 months only, with participants unaware of the incentive until they receive the kit. It is possible that offering incentives at 12 months could influence self-reported behavioural outcomes, but this would be equal in both groups. The incentive could not influence whether or not participants had an STI at 12 months.

Modern mobile phones offer an array of options in which to deliver intervention content. For example, content can be delivered through app instant messaging, videos and audio and can involve bidirectional interactive content. The main trial will evaluate the content of the safetxt intervention messages that could be delivered by alternative modes in the future, such as instant messaging or private social media messaging, should the content be shown to be effective.

The sample size of the main trial is 5000 participants. Of the pilot trial participants with an infection at baseline, 20% (18/89) had a positive chlamydia test at 12-month follow-up. Based on this and previous studies,^3^ we estimated the event rate (cumulative incidence of STI at 12 months) to be 20%. The trial is designed to detect a reduction in STI from 20% to 16% (RR 0.8). Taking into account the 2% contamination in the pilot trial, 5000 participants are required to detect this reduction with 90% power, allowing for 20% loss to follow-up.

## Conclusions

Our pilot trial has demonstrated that recruitment, randomisation, intervention delivery and follow-up were successful. A randomised controlled trial of the safetxt intervention is feasible and is now ongoing.
