# Supplementary material for: Safetxt: a pilot randomised controlled trial of an intervention delivered by mobile phone to increase safer sex behaviours in young people
Source: BMJ Open. 2016 Dec 23;6(12):e013045. doi: 10.1136/bmjopen-2016-013045 (PMC5223743; doi:10.1136/bmjopen-2016-013045)
Supplement: supplementary file [file bmjopen-2016-013045supp1.pdf]

Supplementary file 1. Message frequency

Time post randomisation	Intervention				Control
	Women Positive	Men Positive	Women negative	Men Negative	
	Number of messages				
Week 1	16	17	7	9	1
Week 2	8	8	8	7	0
Week 3	7	6	5	4	0
Week 4	6	5	5	4	1
Month 2	9	9	9	9	1
Month 3	2	2	2	2	1
Month 4	2	1	2	1	1
Month 5	1	1	1	1	1
Month 6	5	5	1	1	1
Month 7	2	2	5	5	1
Month 8	1	1	2	2	1
Month 9	1	1	1	1	1
Month 10	1	1	1	1	1
Month 11	1	1	1	1	1
Month 12	1	1	1	1	1
