# Supplementary material for: Safetxt: a pilot randomised controlled trial of an intervention delivered by mobile phone to increase safer sex behaviours in young people
Source: BMJ Open. 2016 Dec 23;6(12):e013045. doi: 10.1136/bmjopen-2016-013045 (PMC5223743; doi:10.1136/bmjopen-2016-013045)
Supplement: supplementary file [file bmjopen-2016-013045supp2.pdf]

Supplementary file 2. Example intervention and control messages

<u>Intervention</u>

Treatment, informing partners, and adherence

Most people who have an infection don’t know. Your partner(s) could be infected so it’s important to tell them that they need treatment too.

"I talked to my friend and it turned out she’d had it. And so had quite a few others I knew." Text 2 to hear more.

Safer sex behavior support

Think back to a time (or times) when you had sex without a condom. Ask yourself how you could you do things differently next time.

If you’re new to condoms, using them can be tricky at first but it gets a lot easier with practice. Visit LINK for tips on how to use them.

<u>Control</u>

Your participation in the texting study will help us understand more about what kind of help will improve young people's sexual health.

Taking part in the texting study is a way to help you be actively involved in things that affect your life. Thank you for taking part.
