# Supplementary material for: Safetxt: a pilot randomised controlled trial of an intervention delivered by mobile phone to increase safer sex behaviours in young people
Source: BMJ Open. 2016 Dec 23;6(12):e013045. doi: 10.1136/bmjopen-2016-013045 (PMC5223743; doi:10.1136/bmjopen-2016-013045)
Supplement: supplementary file [file bmjopen-2016-013045supp3.pdf]

Texting Sexual Health
Pilot Protocol
v8, 17.07.2013

Project title ... 3

Research objectives ... 3

Background ... 3
- Existing research ... 3
- Justification for this research ... 3

Research methods ... 4
- Design ... 4
- Inclusion criteria ... 4
- Exclusion criteria ... 4
- Recruitment and consent ... 4
- Allocation ... 4
- Protecting against bias ... 4
- Intervention & Control groups ... 5
  - Intervention ... 5
  - Control ... 5
- Message delivery ... 5
- Outcomes ... 5
  - Primary outcomes ... 5
  - Secondary outcomes ... 6
- Data collection ... 6
- Chlamydia sample ... 7
  - Collection ... 7
  - Lab processing ... 7
- Follow up ... 7
  - Incentives ... 7
- Sample size ... 8

Qualitative interviews ... 8

Analyses ... 8

Ethical arrangements ... 9
- Ethical approval ... 9
- Informed consent ... 9
- Participants' rights ... 9
- Participants' safety ... 9
- Retention of trial documentation ... 10

Project timetable and milestones ... 10

Research governance ... 10

Trial management group 10
Trial Steering Committee 10
Service users 10
Data monitoring and ethics committee 10

References 10

Project title

Can text messages increase safer sexual health behaviours in young people? Pilot Trial

Research objectives

To conduct a pilot randomised controlled trial of a mobile phone based intervention to promote safe sexual health behaviour in young people.

- To identify the most effective recruitment processes and assess recruitment rates;
- To test all trial procedures and methods;
- To determine how many trial participants can be followed up at 12 months using the most up to date evidence on methods to achieve optimal follow up;
- To test the acceptability and appropriateness of the intervention when delivered to participants' mobile phones;
- To report the intervention effects on sexual risk behaviours and STI infection;
- To provide prevalence estimates for sexual risk behaviours and Chlamydia reinfection rates at 12 months in the control group with the aim of informing the sample size calculation for the main trial

Background

Existing research

Results from the txt2stop smoking cessation trial (led by Dr Cari Free) show that text messaging interventions can sustain health behaviour change in the long term.[1] The mobile phone text message based intervention doubled biochemically verified smoking cessation at six months: 10.7% of txt2stop participants versus 4.9% of those in the control group that did not receive the intervention. Other recent trials show that text messaging interventions have been effective in increasing adherence to antiretroviral medication and health workers' adherence to malaria treatment guidelines.[2,3] Thus, mobile phone text messaging has the potential to be a powerful way of influencing behavioural change.

In our systematic review of randomised controlled trials of mobile technology based interventions designed to improve health or health care services there were three trials employing short message service (SMS)/ text messages designed to improve sexual health (Lim et al, 2007; Delamere et al, 2006, Menon-Johansson et al, 2006;).[4,5,6,7] Lim and colleagues reported that mobile phone based sexual health interventions can increase discussion of sexual health with a health care professional by threefold and over a doubling in STI testing.[5] Delamere and colleagues' small trial reported no statistically significant effects on unprotected sex and the number of new sexual partners.[6] Menon-Johansson and colleagues demonstrated that conveying Chlamydia results by text message can reduce the time from test result to diagnosis and treatment, but had no effect on the time from first contact to treatment.[7] Whilst some trial results look promising, to date, the effects of text messaging on key safer sex behaviours, including: telling your partner about your infection, correctly following treatment advice, obtaining STI testing for yourself and your partner(s) prior to unprotected sex and condom use, has not been reliably established.

Justification for this research

Support via text message is likely to be acceptable to young people and might increase safer sexual health behaviours. Mobile phones are able to provide confidential and non-judgemental support, which is essential for a sexual health intervention.[8] Interactive support can be delivered at any time, in any location, thus ensuring privacy, which is especially important for young people. Behaviour change techniques used in effective face-to-face interventions can be modified for delivery via text message.[9,10] The content can be personalised for different genders and ethnic groups.

Our intervention has been developed by building on our previous work and experience in: developing an effective text messaging intervention, conducting systematic reviews and content analysis of mobile phone text messaging and condom promotion interventions and developing preliminary content for a mobile phone intervention promoting sexual health.

Research methods

Design

A pilot randomised controlled trial. This will allow us to assess important factors needed to design the main trial, such as recruitment rates and how many people we can follow up at 12 months. Participants will be randomly allocated to receive personalised text messages over their mobile phones providing support to promote sexual health or control messages providing information about trial participation.

Inclusion criteria

- Chlamydia screening programme participants. These can either be young people with a positive Chlamydia test result and/or young people who have had unsafe sex in the last year (more than one partner and at least one occasion of unprotected sex)[11]
- Owner of a personal mobile phone (not a shared work phone)
- Aged 16 to 24

Exclusion criteria

- Non-English language speakers will be excluded from the study as the text messages will be delivered in English
- Those that are unable to provide informed consent (e.g. people with severe learning difficulties)

Recruitment and consent

Staff at the local service (sexual health service or pharmacy) will assess potential participants for eligibility: young people who attend a clinic and were tested for Chlamydia (these can be young people with a positive Chlamydia test result and/or young people who have had unsafe sex in the last year i.e. more than one partner and at least one occasion of unprotected sex); owner a personal mobile phone and are aged between 16-24 years. Eligibility will be assessed either when young people are 1) being tested for Chlamydia at a sexual health service, 2) receiving a test result over the phone, or 3) collecting treatment from services or pharmacies.

After this initial approach by the RN based at the clinic, the research staff will check their eligibility and provide potential participants with detailed verbal and written information and give them the opportunity to ask any questions. If the participant agrees to participate, we will ask them to provide consent. This can be done via the trial website, by text message or by filling out a paper based version, according to their preference and computer availability. If they would like more time to consider their participation, we will give them the Study Coordinator's contact information should they have any further questions or would like to participate.

Allocation

Participants can either enter baseline data directly onto the trial website, by filling out a paper based version of the form, or by phoning the Study Coordinator or designated site researcher who will collect the data, according to their preference. After baseline data has been submitted, an independent randomisation will allocate participants to the intervention or control group.

Protecting against bias

Due to the nature of the intervention, participants will be aware of treatment allocation. The intervention will be delivered by computer ensuring that investigators are unaware of the allocation sequence (allocation concealment). The Study Coordinator will have access to treatment allocation information as they will need to monitor the incoming texts and identify intervention participants for the qualitative interview study. Laboratory staff assessing Chlamydia infection and researchers assessing the outcomes will be blind to treatment allocation.

Intervention & Control groups

Intervention

Through our development work, we have created an acceptable and appropriate mobile phone based intervention to promote safer sex amongst young people.

Participants in the intervention group will receive text messages designed to increase safer sex in three ways: first, by urging participants to correctly follow instructions for treatment of sexually transmitted infections, which includes taking treatment, informing partner(s) about infection and abstaining from unprotected sex for seven days after they and their partner(s) have been treated; second, by encouraging those in the target audience to use condoms with new or casual partners; and third, by encouraging participants to obtain testing for sexually transmitted infections for themselves and their sexual partner(s) prior to unprotected sex (sex without a condom). The intervention also includes general safer sex messages e.g. messages targeting consistent and accurate condom use; condom negotiation skills; the relationship between drugs, alcohol and unprotected sex and information on contraception and pregnancy.

Participants can request information and examples of how other young people have addressed the challenges they face in relation to these key areas. Text content also includes sexual pleasure and covers issues such as trust, intimacy and gender roles. The messages are written in a supportive and encouraging style. They are tailored according to gender, Chlamydia status at the time of enrolment and level of sexual risk behaviour to give personally relevant information and feedback. The information, delivered via text messages, is in accordance to guidelines provided by the British Association for Sexual Health and HIV.[12] All intervention participants will receive regular safer sex top up messages for the duration of their involvement in the trial (12 months).

Control

The rationale for inclusion of a control group is so that we can conduct the pilot study in the same way as the planned trial and so that we can assess potential effectiveness of the intervention. Participants allocated to the control group will receive monthly messages about the importance of participating in the trial.

All participants will be free to use any existing services or interventions.

Message delivery

All messages are sent automatically from a large database to an aggregator. The aggregator has contractual agreements with all the mobile phone network operators and will send messages to each to each participant via their network. Incoming messages from participants will be sent to the long code via the networks and aggregator and can be viewed on the computer system. The success of delivery of messages at each step is monitored by the networks, the aggregator and the computer system that generates and receives the messages. A member of the trial team will automatically be notified if there is any failure in the delivery of messages. All participants will be able to set embargoed times when they do not want to receive messages. If participants text a keyword to the long code number, the sending of messages will automatically stop. Alternatively, the participants can call the trial coordinating centre who will arrange for the messages to stop.

Outcomes

Primary outcomes

The primary outcomes relating to the design of the main trial are: recruitment rates (number of people randomised per month) and completeness of follow up.

We will assess the number of young people recruited at the time of 1) testing, 2) provision of test results or 3) treatment collection. We will assess the number of people enrolled face to face from services or opting to enrol on line.

Secondary outcomes

Participants will provide the self-reported secondary outcome data either through the trial website (in a private consulting room at the service or independently), by filling out a paper based version or phoning the Study Coordinator at baseline, 1 and 12 months.

At Month 1 we will assess:

- For participants testing positive for Chlamydia, gonorrhoea or NSU at the time of recruitment:
  - If they took the treatment [Yes/No]
  - If they avoided sex for 7 days after treatment [Yes/No/Unsure]
  - If they told the last person they had sex with before the test that they needed to get treatment [Yes/No/Sort of]
  - If they avoided sex with this person for 7 days after they took the treatment [Yes/No/Not applicable]
- Condom use at last sex [Yes/No/Unsure]
- Sex with someone new since joining the study [Yes/No/Unsure]
- Condom use at last sex with someone new [Yes/No/Unsure]
- Sexually transmitted infection testing for self prior to sex with someone new [Yes/No/Unsure]
- Participant's report if their last new partner was tested for sexually transmitted infection prior to sex with them [Yes/No/Unsure]
- Number of sexual partners since joining the study [0/1/2+]
- Number of text messages read [All/Some/None]

At Month 3 we will assess:

- Infection (urine test for men and urine or self taken vulvo-vaginal swab for women, according to their preference) [Positive/Negative]

At Month 12 we will assess:

- Infection (urine test for men and urine or self taken vulvo-vaginal swab for women, according to their preference) [Positive/Negative]
- For participants testing positive for Chlamydia, gonorrhoea or NSU after joining the study:
  - If they took the treatment [Yes/No]
  - If they avoided sex for 7 days after treatment [Yes/No/Unsure]
  - If they told the last person they had sex with before the test that they needed to get treatment [Yes/No/Sort of]
  - If they avoided sex with this person for 7 days after they took the treatment [Yes/No/Not applicable]
- Condom use at last sex [Yes/No/Unsure]
- Sex with someone new since joining the study [Yes/No/Unsure]
- Condom use at last sex with someone new [Yes/No/Unsure]
- Sexually transmitted infection testing for self prior to sex with someone new [Yes/No/Unsure]
- Participant's report if their last new partner was tested for sexually transmitted infection prior to sex with them [Yes/No/Unsure]
- Number of sexual partners since joining the study [0/1/2+]
- Number of text messages read [All/Some/None]
- Car accident where the participant was the driver in the past 12 months [Yes/No]

Data collection

The eligibility data we will collect is: Positive Chlamydia test result in the past week; (if Yes) Whether and when they collected the treatment; sex with more than one person in the last year; sex without a condom in the last year; own a personal mobile phone (not a shared work phone); aged 16 to 24; agree to give a urine or self taken vaginal swab test for Chlamydia at 3 and 12 months and if they prefer to give the sample by post or at the clinic.

Participants can either enter baseline data directly onto the trial website, by filling out a paper based version of the form, or by phoning the Study Coordinator or designated site researcher who will collect the data, according to their preference. Data collected via the paper based version or over the phone will be entered by the Study Coordinator or site researcher.

We will collect the following contact details: first name; surname; main mobile phone number; alternative phone number; email address; alternative email address; primary postal address; alternative postal address; name and contact details of someone to contact if we can't reach the participant and their relationship with this person (optional). And we will also collect demographic data: date of birth; gender; ethnicity; sexual orientation. We will also ask all participants what times they do not want to receive messages and whether they prefer to test for Chlamydia via postal test kit or by attending the clinic they were last tested.

The baseline questionnaire will assess:

- Condom use at last sex [Yes/No/Unsure]
- Condom use at last sex with someone new [Yes/No/Unsure]
- Sexually transmitted infection testing for self prior to sex with someone new [Yes/No/Unsure]
- Participant's report if their last new partner was tested for sexually transmitted infection prior to sex with them [Yes/No/Unsure]
- Number of sexual partners in the last 12 months [0/1/2+]

Self reported follow up data at 1 & 12 months will be collected by the method the participant agrees to at enrolment. Objective data regarding infection will be collected at 3 & 12 months by post or through the local service.

Chlamydia sample

Collection

At the 3 and 12 month follow up points, the Study Coordinator will send Chlamydia postal test kits to all participants who indicated a preference for this mode of testing on the enrolment questionnaire. The kits will initially be sent to participants' primary address. The kits will contain instructions on how to collect the sample and how to return the samples to the lab (return packaging and postage provided). We will send urine kits to men and we will send women either a urine or swab test. If participants indicated that they preferred to attend the clinic for testing, the Study Coordinator will liaise with staff at the local service and the participant to facilitate testing.

Lab processing

The lab will process all returned samples and the results will be uploaded onto a secure website created for the trial.

Follow up

Previous trials of behaviour change interventions designed to prevent STIs have achieved 80% follow up for Chlamydia testing.[13] In our previous trial of a text messaging intervention (the txt2stop smoking cessation trial) we used evidence based methods identified in systematic reviews in order to minimise losses to follow up.[14] We will use similar methods in this trial to minimize losses to follow up such as providing incentives to all responders, using pre-notification and contacting non-responders using phone call, text message, email and post. The Study Coordinator will verify participant addresses at enrolment or shortly after, and attempt to contact participants who have provided an incomplete or unknown address.

Incentives

To maximise response rates, we will provide an unconditional (sent with the first questionnaire or Chlamydia test kit) incentive of £5 at each request for follow up as a thank you for participants' time.[1] We will provide £20 on return of each Chlamydia sample.

Participants will be informed about the £5 for each follow up request at enrolment and will be informed of the £20 for Chlamydia sample return at the three and 12 month follow up point. The rationale for this is so that participants join the study because they want to participate, not just for the incentive.

Cash incentives will be provided as follows:
Month 1: Questionnaire; £5 unconditional incentive
Month 3: Chlamydia sample; £5 unconditional; £20 on return
Month 12: Questionnaire & Chlamydia sample; £5 unconditional for each; £20 on return of Chlamydia sample

[1]Edwards PJ, Roberts I, Clarke MJ, DiGuiseppi C, Wentz R, Kwan I, Cooper R, Felix LM, Pratap S: Methods to increase response to postal and electronic questionnaires. Cochrane Database Syst Rev 2009, 3:MR000008.

Sample size
The aim of the pilot trial is to estimate the likely rate of recruitment and rate of follow-up at 12 months to assess the feasibility of the main trial. If losses in the main trial were 20% of participants recruited, our estimate of loss to follow up would be within 6% of this value (i.e. a 95% confidence interval from 14% to 26%) using a pilot sample size of 200 participants. If losses were 10%, our estimate of loss to follow up would be within 4% of the true rate.

Qualitative interviews
We will conduct qualitative interviews with 25 participants two to three weeks after they enrolled. As the interview asks participants to comment on their views on the texts messages, the interview will be conducted shortly after they received the messages in order to minimize problems with recall. The interviews will follow a semi-structured topic guide, which aims to find out about their experiences of the intervention and recommendations for improvement. If the participant indicates at enrolment that they agree to be contacted about being interviewed, the Study Coordinator will telephone participants receiving the intervention to ask if they would be willing to be interviewed regarding their experiences. Participants will be sampled so they vary according to their age, gender, experience of STI and the geographical area they live (urban/ rural). Potential participants will be given verbal and written information about the study and we will obtain informed written consent either by email or text message. According to participants' preference, we will conduct the interviews by telephone or in person at a time and place (e.g. local service) convenient to them. We will give participants £20 cash for completing the interview. We will conduct a shorter follow up interview around 4 months after enrolment, after we have requested the Chlamydia sample at three months.

Analyses
We will estimate follow up at 12 months with a 95% confidence interval. For all other outcome measures we will estimate the relative risk with a 95% confidence interval or mean difference and 95% confidence intervals. All analyses will be based on the intention-to-treat principle. Pearsons chi-squared test will be used to assess differences between intervention and control groups at the 5% level of significance. This is a behavioural intervention unlikely to produce adverse effects, so analysis by the research team will be undertaken once, at the end of the trial.

We will also analyse:
- Delivered and requested texts
- Types of messages sent back
- The timing of responses
- Frequency of responses to particular messages

The following are key metrics that we will use to determine if the pilot trial methods are sufficiently robust to warrant applying for funding for a main trial:

- 93% or higher of messages sent from the aggregator successfully delivered to participants mobile phones (data provided by the aggregator)
- Completed follow up of 80% or higher for the primary outcome for the main trial
- Recruitment of 200 participants within 3 months

The qualitative interviews will be audio recorded and transcribed. We will develop a provisional coding framework and two people will code the transcripts according to the framework. We will search for deviant cases and interviews will be conducted until saturation. We anticipate that about 25 interviews will be required. We will describe the key themes and recommendations. We will use the findings to inform any necessary adjustments to the intervention after the pilot trial. We will write up the results of the qualitative interview study and submit the paper for publication in a peer reviewed academic journal.

Ethical arrangements

Ethical approval

We are applying to Surrey for ethical approval (REC reference 13/LO/1001). The lead R & D site for the trial is Kings College Hospital NHS Foundation Trust and the two additional research sites are Cambridgeshire Community Services NHS Trust and Central Manchester University Hospitals NHS Foundation Trust, from both of whom we are seeking R&D approval.

Informed consent

Participants will be provided with study information and given the opportunity to ask questions. For participants joining the trial, the recruiting staff will check that participants understand that they may or may not be allocated to receive the intervention prior to entry into the trial. They will also check that participants are aware that consenting to participate means that we will be asking for a Chlamydia sample at 3 and 12 months.

Participants' rights

Participants will be able to contact the trial co-ordinating centre by text message to the long code number or by telephone call. Acting on participants' requests to withdraw from the trial, participants' status will be changed to 'withdrawn' on the web based data entry form. This will automatically result in the text messages stopping and the withdrawer being excluded from lists of participants due follow up. Participants will be able to stop text messages, but choose to continue with the trial follow up. Personal details will be stored on a password protected computer held on a secure server at the London School of Hygiene & Tropical Medicine. This information will be stored separately from any anonymised research data, and will be deleted at the end of the study. Participant mobile phone numbers, but no other personal details, will be stored in the operational system used to send out the text messages. No positive test results or personally identifying information will be sent by text message to the participants. Participants will be informed about any positive Chlamydia test results by telephone call and will be advised how to obtain treatment through their local service.

Participants' safety

The intervention provides support and is unlikely to cause any harmful effects. Even small changes in sexual health behaviour will outweigh any plausible risks from using mobile phones. The support might make some participants aware that they are in abusive sexual relationships. We will provide clear information about how to access appropriate counselling services for people in abusive relationships; at the time of recruitment, we will provide a general list of help lines they can contact, including help lines offering support for people experiencing violence. It is possible that some participants will have partners (or others they may not want to see the messages) who may look at the participants' text messages without the participant knowing. We will collect data regarding whether participants were aware of this occurring and the consequences (for example, an argument, a constructive discussion, violence).

Road traffic accidents are rare, but the only demonstrated hazard of text messaging. There was no evidence of any increase in road traffic accidents in our previous trial sending text messages to 5,800 participants,

but we will collect data regarding involvement in road traffic accidents. We will advise participants not to text whilst driving.

Retention of trial documentation

We will retain trial documentation for 10 years.

Project timetable and milestones

By month 6 (September 1st 2013)

- Intervention text messages fully user tested and finalized
- Completed and user tested trial data base and randomisation program
- Completed and user tested IT program, systems and interfaces for the intervention delivery

By month 12 (March 1st 2014)

- Recruitment to the pilot trial completed (the target is to recruit 200 participants over 3 months)
- Qualitative interviews completed

By month 24 (March 1st 2015)

- Analysis of the qualitative interviews completed and a paper submitted for publication based on the qualitative data
- Database for the pilot trial closed
- Analysis of the pilot trial results completed.
- Follow up rates at 1 month and 12 months for self-reported data 80% or higher
- Follow up rates for Chlamydia testing 80% or higher
- Paper submitted for publication based on the pilot trial results

Research governance

Trial management group

Permanent members of the group will be Dr Caroline Free, Dr Rebecca French and Ms Ona McCarthy. Co-applicants will be invited to join the group during the phases of the research where their expertise is required for example Dr Susan Michie, Dr Kaye Wellings and Dr Graham Hart will be invited to the group during the intervention development phase.

Trial Steering Committee

This will comprise an independent member (Tess Harris), a statistician (Andrew Copas), and chair (Dr Pippa Oakshott) and one Service User. It will meet every 6 months after the pilot trial has commenced.

Service users

The Trial Steering Committee will include two young people. We will provide young people involved in the research with the study results.

Data monitoring and ethics committee

This is a pilot trial so we are not planning a DMEC. The TSC will take on the ethical roles normally undertaken by the DMEC. We have contacted the HTA who have agreed to this.

References

[1] Free C, Knight R, Robertson S, Whittaker R, Edwards P, Zhou W, et al. Smoking cessation support delivered via mobile phone text messaging (txt2stop): a single - blind, randomised trial. Lancet. 2011 Jul 2;378(9785):49 - 55

[2] Zurovac D, Sudoi RK, Akhwale WS, Ndiritu M, Hamer DH, Rowe AK, et al. The effect of mobile phone text - message reminders on Kenyan health workers' adherence to malaria treatment guidelines: a cluster randomised trial. Lancet. 2011 Aug 27;378(9793):795 - 803.

[3] Lester RT, Ritvo P, Mills EJ, Kariri A, Karanja S, Chung MH, et al. Effects of a mobile phone short message service on antiretroviral treatment adherence in Kenya (WelTel Kenya1): a randomised trial. Lancet. 2010 Nov 27;376(9755):1838‐45.4 Bennett DA, Emberson JR. Text messaging in smoking cessation: the txt2stop trial. Lancet. 2011 Jul 2;378(9785):6‐7.

[4] Free C, Ian R, Abramsky T, Fitzgerald M, Wensley F. A Systematic Review of Randomised Controlled Trials of Interventions Promoting Effective Condom Use. J Epidemiol Community Health. 2009 Oct 12.

[5] Lim MSC, Hocking JS, Aitken CK, Jordan LC, Fairley CK, Lewis JA, et al. A Randomised controlled trial of the impact of email and text (SMS) messages on the sexual health of young people. Sexual Health. 2007;4.

[6] Delamere S, Dooley S, Harrington L, King A, Mulcahy F. Safer sex text messages: Evaluating a health education intervention in an adolescent population. Sexually Transmitted Infections. 2006;82:A27.

[7] Menon-Johansson AS, McNaught F, Mandalia S, Sullivan AK. Texting decreases the time to treatment for genital Chlamydia trachomatis infection. Sex Transm Infect. 2006 Feb;82(1):49‐51.

[8] Thomas N, Murray E, Rogstad KE. Confidentiality is essential if young people are to access sexual health services. Int J STD AIDS. 2006 August 1, 2006;17(8):525‐9.

[9] Free C, Whittaker R, Knight R, Abramsky T, Rodgers A, Roberts IG. Txt2stop: a pilot randomised controlled trial of mobile phone‐based smoking cessation support. Tob Control. 2009 Apr;18(2):88‐91.

[10] Abraham C, Michie S. A taxonomy of behavior change techniques used in interventions. Health Psychol. 2008 May;27 (3):379‐87.

[11] Wellings K, Nanchahal K, Macdowall W, McManus S, Erens B, Mercer CH, et al. Sexual behaviour in Britain: early heterosexual experience. Lancet. 2001 Dec 1;358(9296):1843-50.

[12] Clutterbuck D, Flowers P, Fakoya A, Barber T, Wilson H, Nelson M, et al. The United Kingdom National guideline on safer sex advice. The Clinical Effectiveness Group of the British Association for Sexual Health and HIV (BASHH) and the British HIV Association (BHIVA)2011.

[13] Shain RN, Piper JM, Newton ER, Perdue ST, Ramos R, Champion JD, et al. A randomized, controlled trial of a behavioral intervention to prevent sexually transmitted disease among minority women. N Engl J Med. 1999 Jan 14;340(2):93-100.

[14] Edwards P, Roberts I, Clarke M, DiGuiseppi C, Pratap S, Wentz R, et al. Increasing response rates to postal questionnaires: systematic review. BMJ. 2002 May 18;324(7347):1183.
